# Cyclodepsipeptides: Isolation from Endophytic Fungi of *Sarcophyton ehrenbergi* and Verification of Their Larvicidal Activity via In-Vitro and In-Silico Studies

**DOI:** 10.3390/md20050331

**Published:** 2022-05-18

**Authors:** Abdel Nasser B. Singab, Nada M. Mostafa, Yasmin A. Elkhawas, Eman Al-Sayed, Mokhtar M. Bishr, Ahmed M. Elissawy, Mohamed S. Elnaggar, Iten M. Fawzy, Osama M. Salama, Yi-Hong Tsai, Fang-Rong Chang

**Affiliations:** 1 Department of Pharmacognosy, Faculty of Pharmacy, Ain-Shams University, Cairo 11566, Egypt; nadamostafa@pharma.asu.edu.eg (N.M.M.); em_alsayed@pharma.asu.edu.eg (E.A.-S.); aelissawy@pharma.asu.edu.eg (A.M.E.); Mohamed.s.elnaggar@pharma.asu.edu.eg (M.S.E.); 2 Center of Drug Discovery Research and Development, Ain-Shams University, Cairo 11566, Egypt; 3 Department of Pharmacognosy and Medicinal Plants, Faculty of Pharmacy, Future University in Egypt, Cairo 11835, Egypt; yasmien.alaa@fue.edu.eg (Y.A.E.); osalama@fue.edu.eg (O.M.S.); 4 Department of Research and Development, Mepaco Co., Sharkeiya 11361, Egypt; mbishr_2000@yahoo.com; 5 Department of Pharmaceutical Chemistry, Faculty of Pharmacy, Future University in Egypt, Cairo 11566, Egypt; Iten.mamdouh@fue.edu.eg; 6 Graduate Institute of Natural Products, College of Pharmacy, Kaohsiung Medical University, Kaohsiung 80708, Taiwan; lyph0719@hotmail.com; 7 Department of Pharmacy and Master Program, Collage of Pharmacy and Health Care, Tajen University, Pingtung County 90741, Taiwan; 8 Department of Medical Research, Kaohsiung Medical University Hospital, Kaohsiung 80708, Taiwan; 9 Drug Development and Value Creation Research Center, Kaohsiung Medical University, Kaohsiung 80708, Taiwan; 10 Department of Marine Biotechnology and Resources, National Sun Yat-sen University, Kaohsiung 80424, Taiwan

**Keywords:** chitinase, *Culex pipiens*, docking, endophytes, *Sarcophyton ehrenbergi*, protease, phenoloxidases, lipase

## Abstract

*Culex pipiens* mosquitoes are vectors to many viruses and can transmit diseases such as filariasis and avian malaria. The present study evaluated the larvicidal activity of marine-derived endophytic fungi *Aspergillus nomius* and *Aspergillus flavus* from the soft coral *Sarcophyton ehrenbergi* along with two known cyclodepsipeptide compounds, scopularide A (**1**) and B (2), isolated from *A. flavus* extract, against third-instar larvae of *C. pipiens*, using distilled water as a negative control and toosenedanin as a positive control. The structures of the isolated compounds were confirmed by various spectroscopic analyses. The lethal concentrations (LC_50_ and LC_90_) were calculated by probit analysis. Scopularide A was the most potent after 96 h treatment, with LC_50_ and LC_90_ values of 58.96 and 994.31 ppm, respectively, and with 82.66% mortality at a concentration of 300 ppm. To unravel the biochemical mechanism of the tested extracts and compounds, their effects against protease, chitinase, phenoloxidases and lipase enzymes from the whole-body tissue of *C. pipiens* were evaluated after 72 h treatment at LC_50_ dose. Superior activity was observed for *A. flavus* extract against all tested enzymes. A molecular docking study was conducted for scopularide A and B on the four tested enzymes, to further verify the observed activity. Results revealed good binding affinities for both compounds as compared to the docked ligands, mainly via a number of hydrogen bonds. This was the first study to report the isolation of endophytic fungi *A. flavus* and *A. nomius* from the marine soft coral *S. ehrenbergi.* The endophytic fungal extract of *A. flavus* was found to be a promising source for a natural larvicidal agent against *C. pipiens* populations.

## 1. Introduction

Insects are vital organisms that have been shown to benefit humans in a variety of ways, including environmental stability, agriculture, and nutrition. Several other insects, on the other hand, are dangerous in terms of causing health problems, crop loss, or even pollution. Pests and vectors are the most harmful organisms, causing major health and productivity problems. Insect pests cause damage to crops by feeding on plants or stored cereals. Increased pest attacks frequently result in lower productivity and, as a result, a weaker economy [1]. Outbreaks of these pests frequently endanger worldwide food safety and, as a result, raise health concerns [2]. Aside from pests, vectors, which include mosquitoes, are another important group of insects that have a major impact on healthcare systems. Mosquitoes are the primary vectors of pathogens such as Zika virus and malarial and dengue virus in tropical and subtropical countries such as India, South Africa, and Brazil [3]. According to WHO, mosquitoes are considered the number one “public enemy” [4]. Mosquito-borne diseases are common in more than one hundred countries across the world, infecting over four billion people every year worldwide [5].

These vectors can only be controlled by manmade insecticides such as organochlorines, organophosphates, carbamates, and DDTs. The misuse of these synthesized insecticides has led to environmental problems, in addition to the emergence of resistant variants [6,7]. To ensure the sustainability of the ecosystem, components with low or no toxicity to non-target organisms should be used [8]. Natural products derived from fungi or other biological sources are inexpensive, environmentally friendly, and play an important role in alleviating these types of problems [9,10]. Natural products provide effective alternatives and promising sources for many bioactivities [11,12,13,14,15]. Discovering larvicides from marine sources with biodiversity may be another approach to mosquito control. Natural-originated and -derived larvicides contain a mixture of metabolites, which act concurrently on both physiological and behavioral processes and may exert actions different from single synthetic larvicides, which may lead to unexpected resistance [16]. Identifying efficient as well as ecofriendly bio-larvicides is crucial for continued efficient mosquito management. Marine organisms provide a reservoir of a vast number of organisms, such as bacteria or fungi, that can live symbiotically within their tissues [17,18]. The bioactive metabolites produced by some seaweeds showed a wide range of biological activities, from insecticidal against arthropods to neurologically active in humans [19,20].

Endophytes are microorganisms, generally fungi and bacteria, that spend part or all their lives in the host marine creature [21]. In healthy and fine tissue, endophytic microorganisms can be discovered. Owing to the biotechnological interest of fungal endophytes as biological control agents, metabolites, and genetic vectors, endophytes have been observed as an excellent reservoir of secondary bioactive metabolites [22]. They are able to defend their hosts against several biotic and abiotic aspects, e.g., the attack of pathogens, insects, and herbivores [23].

Molecular docking is considered as a mockup technique that discovers the best fitting pose between ligands and the active sites of the specific targets. This procedure includes the arrangement of the 3D coordinate space on the binding site of the target, calculation of the binding affinity of the formed complex, and elaboration of the subsequent orientation of a molecule on the ligand’s binding site. The largest magnitude negative number signifies the sensitivity of binding affinity, representing the most satisfactory conformation of the formed complex [24,25,26,27]. Here, we reveal the isolation and larvicidal activity of two cyclodepsipeptides, scopularide A and B, along with the crude extracts, from the culture rice media of both endophytic *A. flavus* and *A. nomius*, isolated from *Sarcophyton ehrenbergi*. This is the first study demonstrating the mosquito larvicidal activity of Egyptian marine endophytic fungi.

## 2. Results

### 2.1. Compound Identification

**Figure 1:**
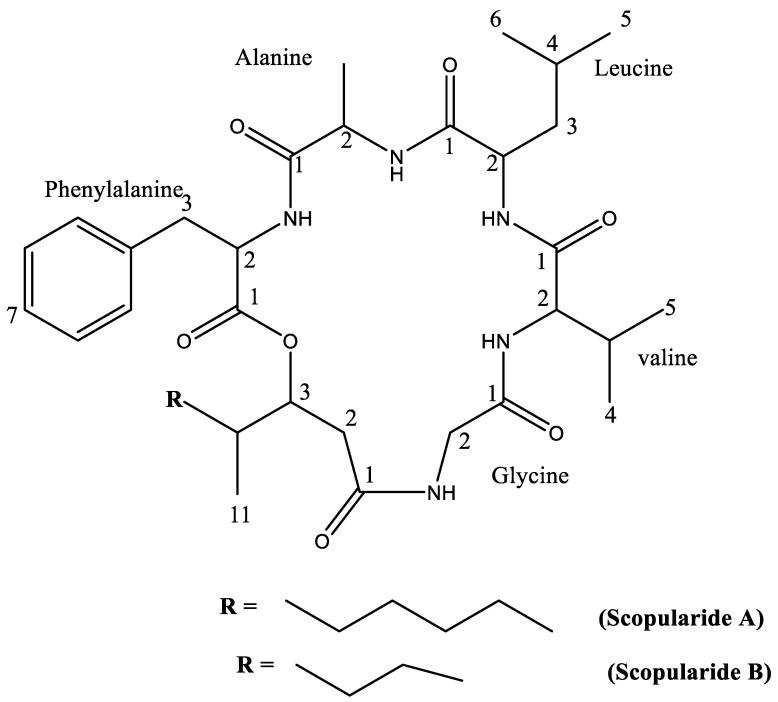
Chemical structure of isolated cyclodepsipeptides.

Compound **1** was isolated as thin off-white crystals, m.p. 228–232 °C; the purity of the isolated crystals was indicated by the sharp melting point observed. Pure compounds have sharp melting points, which, according to Dent [28], are compounds with melting points within a temperature range of 5 °C. NMR data of compound **1** (Table 1 and Table S1) were in accordance with those reported for the cyclodepsipeptide, scopularide A [29]. Compound **2** was isolated as white amorphous powder. Its NMR data (Table 2 and Table S2) were in accordance with the reported data for scopularide B [29]. The structures of the isolated compounds are presented in Figure 1.

### 2.2. Larvicidal Activity

To assess the mosquito larvicidal activities of the crude extracts, third-instar larvae of *C. pipiens* were used. Crude extracts of *A.* *flavus* and *A.* *nomius* as well as the isolated scopularides A and B were tested against *C. Pipiens*. Table 3 and Table 4 summarize the toxicities of different concentrations of the two isolated compounds and *A. flavus* and *A. nomius* crude extracts at various time intervals (24, 48, 72, and 96 h) and their the LC_50_ values. The mortality of *C. pipiens* larvae was directly proportional to the concentrations of the tested material, i.e., a potential linear relationship between the concentration and mortality percentages exists, also with the extension of the exposure period. The mortality rate after 96 h treatment ranged from 26.66% to 82.66% for scopularide A, 25.33% to 80% for scopularide B, 18.66% to 74.66% for *A. flavus*, and 24% to 78.66 for *A. nomius*. The most potent one was scopularide A as it showed the lowest LC_50_ of 69.96 ppm and 58.96 ppm after 72 and 96 h treatment, respectively. The toxicity index was 100 for both time intervals. Following scopularide A was scopularide B and then *A. nomius* crude extract. The LC_50_ values and toxicity index of the least potent sample, *A. flavus* crude extract, were 102.78 and 94.76 ppm, and 68.10 and 62 ppm, after 72 and 96 h treatments, respectively. Toosenedanin was used as a positive control, with LC_50_ and LC_90_ values of 2.66 and 10.00 ppm after 72 h treatment.

**Figure 2:**
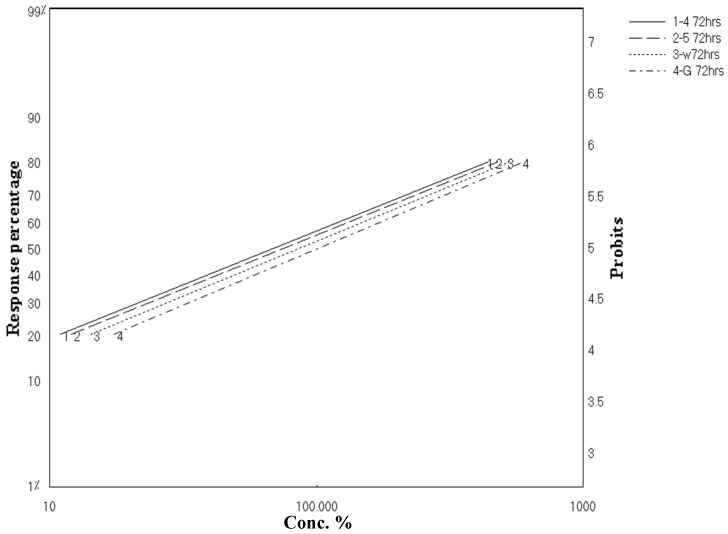
Toxicity regression lines of crude extracts of *A. flavus*, *A. nomius*, and isolated compounds against third-instar larvae of *C. pipiens* after 72 h exposure.

**Figure 3:**
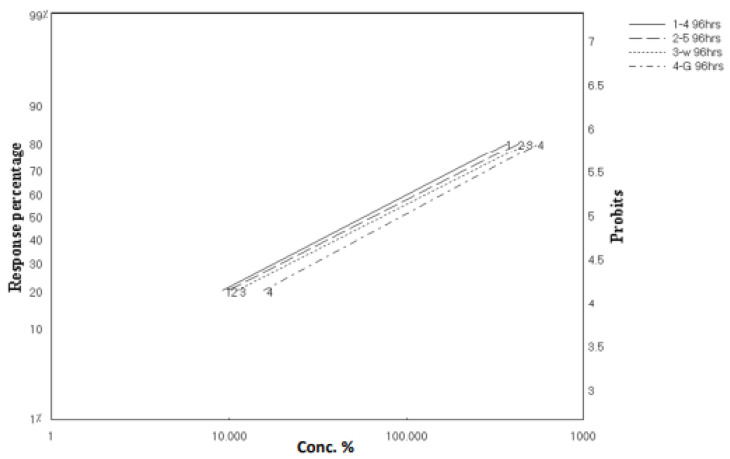
Toxicity regression lines of crude extracts of *A. flavus*, *A. nomius*, and isolated compounds against third-instar larvae of *C. pipiens* after 96 h exposure.

As clarified in Figure 2 and Figure 3, these results revealed parallel toxicity regression lines and indicated that all tested materials possess the same mode of action. This might be able to be predicted because both extracts and the isolated compounds were from the same genus, *Aspergillus* sp. In addition, all tested materials possessed a latent kill effect in which they showed a significant LC_50_ starting from 72 to 96 h.

### 2.3. Biochemical Effects of A. flavus, A. nomius Extracts, and Isolated Compounds against C. pipiens Larvae

#### 2.3.1. Quantitative Analysis (Spectroscopic Analysis)

It was important to investigate the biochemical changes in the activities of some enzymes (protease, chitinase, phenoloxidases, and lipase) from the whole-body tissue of *C. pipiens* larvae after 72 h treatment with LC_50_ of *A. flavus*, *A. nomius* extracts, and isolated compounds.

#### 2.3.2. Determination of Chitinase

Chitinase is a hydrolytic enzyme that breaks down glycosidic bonds in chitin, which is present in the exoskeletons of arthropods. The chitinase activities in the larvae homogenate of *C. pipiens* treated with LC_50_ for 72 h in four examined samples are presented in Table 5. *A. flavus* extract showed an increase in the enzyme activity as 118 ± 8.6 ^a^ mg *N*-acetylglucosamine/min/mg protein, with a percentage change of 9.3%, compared with 108 ± 5.1 ^a^ mg N acetyl glucose amine/min/mg protein in the untreated control group. *A. nomius* extract, scopularide A, and scopularide B showed an insignificant reduction in enzymatic activity of 95 ± 3.2 ^b^, 86 ± 2.5 ^b^, and 82 ± 2.4 ^b^ mg *N*-acetylglucosamine/min/mg protein, with percentage changes of −12.0%, −24.1%, and −20.3%, respectively.

#### 2.3.3. Determination of Phenoloxidases

Phenoloxidase is an enzyme produced by insects as a defense system against bacterial and fungal infections. Enzymatic processes generate superoxide, diphenols, hydrogen peroxide, quinones, and reactive nitrogen intermediates, all of which are necessary for defense. The phenoloxidase activities in the larvae homogenate of *C. pipiens* treated with LC_50_ for 72 h for four examined samples are presented in Table 6. The analysis revealed that phenol oxidase was decreased to 1579 ± 14.5 ^c^ O.D. units × 10^3^/min/mg protein, equal to −11.88%, with *A. flavus* extract, compared with 1792 ± 15.6 ^a^ O.D. units × 10^3^/min/mg protein in the untreated control group, followed by scopularide B, scopularide A, and *A. nomius* crude extract.

#### 2.3.4. Determination of Lipase

The lipase activities in the larvae homogenate of *C. pipiens* treated with LC_50_ for four examined samples for 72 h are presented in Table 7. *A. flavus* crude extract, scopularide A, and scopularide B showed significant increases in enzymatic activity as 4.6 ± 0.22 ^a^, 3.8 ± 0.16 ^b^, and 3.9 ± 0.15 ^b^ μM oleic acid liberated/min/gram body weight, with percentage changes of −52.7%, −26%, and −28.3%, respectively, compared with 3 ± 0.12 ^c^ μM oleic acid liberated/min/gram body weight in the untreated control group.

#### 2.3.5. Determination of Protease

The protease activities in the larvae homogenate of *C. pipiens* treated with LC_50_ for four examined samples for 72 h are presented in Table 8. Both crude extracts, scopularide A and B, showed an insignificant reduction in enzymatic activity as 3235 ± 106 ^ab^, 2873 ± 61 ^c^, 3116 ± 78 ^b^, and 2277 ± 42 ^d^ ɳg protein, compared with 3426 ± 88 ^a^ in the untreated control group, with percentage changes of 5.6%, 16.1%, 9.0%, and 33.5%, respectively.

### 2.4. Molecular Docking Simulation

#### 2.4.1. In Silico Molecular Docking Studies for Larvicidal Enzymatic Activity

##### Chitinase Enzyme

**Figure 4:**
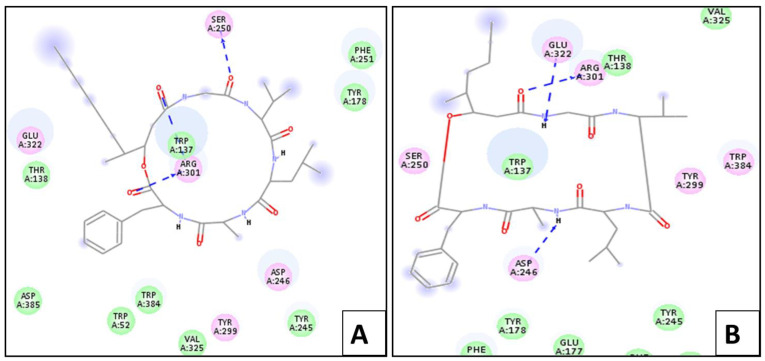
Two-dimensional interaction diagram between scopularide (**A**,**B**) and chitinase.

Upon docking, the ligand showed –(C-docker interaction energy) = −49.23 kcal/mol. It also showed six essential hydrogen bonds with Glu 177, Tyr 245, Asp 246, Arg 301, and Glu 322. On the other hand, scopularide A and B showed –(C-docker interaction energy) of 65.85 Kcal/mol and −59.25 Kcal/mol, respectively, which are relatively better than that of the ligand. The most active conformer of scopularide A showed interaction with chitinase via three hydrogen bonds with Arg 301, Trp 137, and Ser 250, while scopularide B showed a further three hydrogen bonds with Asp 246, Arg 301, and Glu 322, as shown in Figure 4.

##### Lipase Enzyme

When docking was applied, ligand oleic acid showed –(C-docker interaction energy) = −42.73 kcal/mol, while scopularide B displayed –(C-docker interaction energy) = −61.09 kcal/mol and scopularide A failed to display any interaction with lipase. The binding mode of oleic acid with lipase was via three hydrogen bonds between its carboxylic acid group, Ser 83, and Asn 92. Meanwhile, scopularide B showed hydrogen bonds with Asn 92 and Val 203.

##### Protease Enzyme

**Figure 5:**
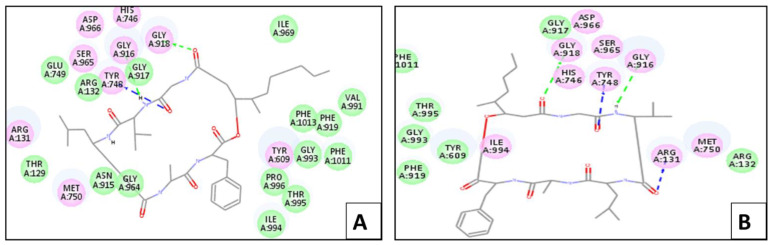
Two-dimensional interaction diagram between scopularide (**A**,**B**) and protease.

The ligand displayed –(C-docker interaction energy) = −99.54 kcal/mol and hydrogen bonds with Arg 132, Tyr 609, Gly 918, and Gly 964. Figure 5 presents the bindings of both scopularide A and B with protease, respectively. Scopularide A, which showed –(C-docker interaction energy) = −65.46 kcal/mol, displayed three hydrogen bonds with Tyr 748, Gly 916, and Gly 918. Meanwhile, scopularide B had –(C-docker interaction energy) = −62.22 kcal/mol and showed four hydrogen bonds with Arg 131, Tyr 748, Gly 916, and Gly 918.

##### Phenoloxidase Enzyme

The ligand *O*-methylated saccharide showed –(C-docker interaction energy) = −33.25 kcal/mol with interaction with phenoloxidase via hydrogen bonds with Thr 318 and Thr 340. Meanwhile, scopularide A docked against phenoloxidase showed −43.68 kcal/mol as –(C-docker interaction energy) and the same two hydrogen bonds, with an interaction similar to that of the ligand with Thr 318 and Thr 340. On the other hand, scopularide B’s interaction was through hydrogen bonds with Thr 318 and Thr 340 and an extra hydrogen bond with Gly 317, and it also showed –(C-docker interaction energy) = −37.79 Kcal/mol.

## 3. Discussion

*C. pipiens* is the most widely dispersed mosquito species in the world. Mosquitoes in Egypt are vectors of malaria, several forms of filariasis, and numerous arboviruses, such as dengue and yellow fever [30]. Thangum and Kathiresan investigated a wide range of marine creatures for insecticidal activity towards mosquito species; one of their research works, published in 1996, was the first case to look into the larvicidal activity caused by marine resources [31]. Bahgat et al. used spinosadas, made from soil Actinomycete, to investigate the environmental risks of employing synthetic insecticides against mosquitoes [32]. There have been numerous studies focused on terrestrial plants to exert mosquito larvicidal activity [33].

Endophytic fungi have been shown to protect their hosts against pests, insects, pathogens, and even domestic herbivores [34]. *A. flavus* and *Penicillium sublateritium*, which live on the host plant, can produce useful metabolites to improve their fitness and fight stress [35]. Biochemical analyses focused on the mosquito larvae’s vital enzymes aided us in understanding the toxicity of substances to mosquito larvae. In contrast to synthetically derived insecticides, naturally derived insecticides are gaining popularity because they do not endanger animals or humans [35]. In view of these results, the purified active compounds as well as endophytic isolated fungi extract found in our studies could be effective in killing mosquito third-instar larvae in an economic and safe manner. Many isolated natural chemical compounds and extracts have been reported for their potential biological activities [36,37,38,39]. Our results showed that isolated scopularide A possessed larvicidal activity, with values of LC_50_ 69.963 and 58.968 ppm after 72 and 96 h treatment, respectively. *A. flavus* crude extract was the most effective one. Through biochemical analyses of certain enzymes to identify the mode of action by which the tested samples exert their activity, it was noticed that changes in the activities of lipase, chitinase, protease, and phenoloxidase enzymes lead to the death of the mosquito larva. These results suggested that the stereochemistry of both compounds plays an important role in their biological activity, which was also confirmed through in silico molecular docking studies. The findings suggest that the endophytic fungus *Aspergillus flavus* could be developed as a natural larvicidal product.

## 4. Materials and Methods

### 4.1. Fungal Material and Identification

The soft coral *Sarcophyton ehrenbergi* was collected in 2015 from Hurghada, Red Sea at 10 m depth, Egypt. It was identified by Dr. Hamada Ali of the National Institute of Oceanography and Fisheries, Hurghada. A voucher sample was kept at Ain Shams University under the voucher sample code of PHG-M-SE-259. The fungal strain was isolated from the coral using the previously described standard technique [40]. Based on DNA amplification and sequencing of the ITS region, the fungus was identified as *A. flavus* (GenBank accession no. Mk969114) [41,42] and the closest relative strain was *A. nomius* (GenBank accession no. MK969116).

### 4.2. Cultivation, Extraction, and Isolation

The strains were grown in 12 1 L Erlenmeyer flasks for 30 days at room temperature under static conditions, using a solid rice medium prepared by autoclaving 100 g rice and 120 mL water at 121 °C for 20 min. Following fermentation, EtOAc (3 × 600 mL) was used to extract the fungal culture in each flask. The obtained crude extract of *A. flavus* (1.2 g) was partitioned between *n*-hexane and 90% aqueous MeOH, giving 850 mg of 90% aqueous MeOH extract. The latter was subjected to vacuum liquid chromatography (VLC) on silica gel 60 eluting with gradient mobile phase (*n*-hexane-EtOAc 100:0 to 0:100, DCM-MeOH 100:0 to 0:100) to give eight fractions (V1-V3). Fraction 3 was subjected to Sephadex LH-20 using MeOH and yielded scopularide A and B, as previously isolated [29,42].

### 4.3. Nuclear Magnetic Resonance (NMR) Spectrometer

Bruker Ascend 400/R (Bruker^®^, AVANCE III HD, 400 MHz, Zürich, Switzerland)—Center for Drug Discovery, Research and Development, Faculty of Pharmacy, Ain Shams University [43,44,45].

### 4.4. Investigation of the Molecular Target Involved in the Larvicidal Activity of the Isolated Compounds

Scopularides A and B were further virtually tested for their activity against chitinase, lipase, protease, and phenoloxidase using Discovery Studio 4.0. The proteins were downloaded from PDB with codes (3CHC, 1GT6, 1NF6, and 6RGG), respectively. All proteins were cleaned, unneeded chains were removed, and hydrogens were added. Simulation using CHARMm forcefield and partial charge MMFF94 was applied and a heavy atom was created. Constraints were turned to be fixed and minimization of proteins took place. Receptor and binding site were identified from the complexed ligand interaction site. For chitinase (3CHC), the crystal structure of Aspergillus fumigatus chitinase B1 in complex with monopeptide ligand -(2S)-2- acetamido- *N*-methyl-5-[[*N*- (methyl carbamoyl) carbamimidoyl] amino] pentanamide- was downloaded from PDB. In lung homogenates from a mouse model of chronic asthma, the peptide in complex was found to inhibit chitinase B1 from *Aspergillus fumigatus* (AfChiB1), human chitotriosidase, and chitinase activity, with potencies ranging from high nanomolar to high micromolar suppression. The linear peptides’ conformations were very comparable to those of the natural product, according to X-ray crystallographic examination of the chitinase inhibitor complexes [46]. Lipase (1GT6) was downloaded from PDB as the S146A mutant of Thermomyces (Humicola) *lanuginosa* lipase in complex with oleic acid [47]. Tricorn protease in complex with Z-Phe-diketo-Arg-Glu-Phe—a trideca-peptide inhibitor modifying the catalytic Ser965 that revealed part of the peptide trapped inside the channel of the beta7 domain—was downloaded from PDB with code (1N6F) [48]. *Photorhabdus laumondii* lectin PLL2 in complex with O-methylated PGL-1-derived disaccharide was downloaded from PDB with code (6RGG). The O-methylated saccharides interacted with two-propeller lectins, PLL2 from the entomopathogenic bacterium *Photorhabdus laumondii* and its homologue PHL from the related human pathogen *Photorhabdusa symbiotica*, in this study. O-methylation is an uncommon sugar alteration with a poorly understood function. Methylated sugars were postulated to constitute a conserved pathogen-associated molecular pattern because of their presence and identification by lectins involved in the immune response. [49]. C-docker was applied between each of the prepared ligands: scopularides A and B and the four minimized proteins. The –(C-docker interaction energy) of the top active conformer out of the resulting 10 and the binding modes were analyzed, visualized, and compared with each of the complexed ligands

### 4.5. Larvicidal Activity

In a series of experiments, the larvicidal activity of the crude extract of *A. flavus*, *A. nomius* as well as the isolated scopularides A and B was assayed against the 3rd-instar larvae of *C. pipiens* using an immersion method as compared to toosenedanin (positive control) according to WHO [4], as follows.

Groups of 25 early 3rd-instar larvae of *C. pipiens* were transferred into test cups, each containing water (100 mL), under laboratory conditions. Each fungal extract was prepared in five different concentrations, with mortality rates ranging from 20% to 90%. Three replicates for each treatment and one for the untreated control (distilled water) were used in the experiment. Every day, abnormal pupae were removed and placed in labeled glass vials containing 70% ethanol.

### 4.6. Method for Larvicidal Activity Using Biochemical Analysis

The insects were prepared in accordance with the guidelines provided by [50]. The insects were mixed in distilled water (50 mg/1 mL). Homogenates were centrifuged at 8000 rpm for 15 min at 2 °C in a refrigerated centrifuge. The deposits were discarded and the supernatants could be stored for at least one week without considerable damage of activity when stored at 5 °C. All experiments contained 3–4 replicates (insect homogenates), and the results of biochemical investigations were combined from triplicate determinations. The data were examined using statistical software and a one-way analysis of variance (ANOVA) (Cohort software, Berkeley). When the ANOVA data were significant, Duncan’s multiple range test (*p* < 0.01) was used to compare the means.

### 4.7. Method for Determination of Total Proteins (Spectroscopic Analysis)

#### 4.7.1. Protein Preparation

Protein solutions were prepared in 0.15 M NaCl according to [51].

#### 4.7.2. Preparation of Protein Reagent

Protein reagent was prepared by dissolving 100 mg of Coomassie Brilliant Blue G−250 in 50 mL 95% ethanol. To this solution, 100 mL 85% (W/V) phosphoric acid was added. The resulting solution was diluted to a final volume of 1 L. For the sample solution (50 μL) for preparation of the standard curve, 50 μL of serial concentrations containing 10 to 100 μg bovine serum albumin was pipetted into test tubes. The volume in the test tube was adjusted to 1 mL with phosphate buffer (0.1 M pH 6.6). Five millimeters of protein reagent were added to the test tube and the contents were mixed by inversion. The absorbance at λ 595 nm was measured after 2 min and before 1 h against a blank prepared from 1 mL of phosphate buffer and 5 mL protein reagent.

### 4.8. Mechanism of Action of Crude Extracts of Both A. flavus and A. nomius and the Isolated Compounds Scopularide A and B in Larvicidal Activity

#### 4.8.1. Chitinase Activity

Substrate preparation: According to [52], colloidal chitin was prepared, as follows. First, 4.0 g of purified chitin powder was suspended in 100 mL water at 4 °C and stirred. Conc. H_2_SO_4_ (30 mL) at 4 °C was added to the suspension. We then filtered the cold chitin solution through glass wool into 1800 mL ice-cold 50% ethanol while stirring. We washed the precipitated chitin with distilled water to pH 5.

Enzyme assay: The reaction mixture was prepared according to [53], with some modifications; we used 1 mL phosphate buffer (0.2 M, pH 6.5), 200 mL 0.5% colloidal chitin, and 200 mL enzyme solution. Enzyme activity was determined after 1.5 h incubation at 37 °C, by boiling the test tube. At 8000 rpm, undigested chitin was sedimented by centrifugation for 15 min. The supernatant was taken for the determination of *N*-acetylglucosamine produced as a result of chitin digestion by chitinase.

Determination of *N*-acetylglucosamine: It was determined by the sensitive method of [54]. The volume of the appropriate aliquot from the supernatant was adjusted to 1 mL with phosphate acetate buffer (0.2 M, pH 6). For each determination, a 1 mL buffer blank and a series of *N*-acetylglucosamine standards (10, 20, 40, 60, and 80 g) in 1 mL buffer were used. Each tube was shaken and heated in a boiling water bath for 10 min, after the addition of 0.3 mL saturated sodium borate solution. The tubes were then transferred rapidly to cold water, and then 8 mL glacial acetic acid was added to each tube. The tubes were shaken and allowed to stand for 30 min at room temperature. The enzyme activity was expressed as μg *N*-acetylglucosamine (NAGA) × 103/min/gm fresh weight.

#### 4.8.2. Lipase Activity

Lipase activity was investigated by a slight modification of the procedure of [55]. The approach worked by measuring the decrease in the ester content of triolein as a substrate.

Substrate: The lipid emulsion composed of 4 g triolein, 7.74 g triton, 0.22 g CaCl_2_, and 0.234 g NaCl made to 100 mL with 0.2 mol/L of tris buffer (pH 7.5).

Assay method: 1 mL substrate emulsion, sample solution 100 µL, and 0.4 mL 0.2 M tris buffer (pH 7.5), incubated at 35 °C for exactly 5 min. After incubation, iso-propanol 4 mL and 1M H_2_SO_4_ 2 mL mixture were added. The reaction products were extracted in 5 mL *n*-heptane. The mixture was allowed to stand for 5 min after vigorous stirring. One mL of *n*-heptane layer was transferred to the test tube and the optical density of the ester content was determined as compared to the standard. Lipase activity was expressed as the number of µM oleic acid liberated/min/gram body weight.

#### 4.8.3. Proteolytic Activity

Tatchell et al. described a method for determining proteolytic activity, which we adopted with some modifications [56]. During 1 h incubation at 30 °C, the increase in free amino acids separate from substrate albumin was measured. Reaction mixture: insect homogenate 100 µL, 0.1 M phosphate buffer 1 mL (pH 8), and 0.5% bovine serum albumin 100 µL. First, 1.2 mL 20% TCA was added to end the reaction. The mixture was centrifuged at 3000 rpm for 20 min after standing for 15 min. Then, the supernatant was used for measuring the quantity of the produced amino acids. Amino acids were colorimetrically assayed by ninhydrin reagent according to the method described by [57]. The reaction mixture: supernatant 100 µL, ninhydrin-citrate (pH 5.5) 1.9 mL, 0.5 M citrate buffer (pH 5.5) 0.2 mL, and 1.2 mL glycerol. In a boiling water bath for 12 min, the mixture was heated and cooled by tap water. The developed color was measured at λ 570 nm, zero adjustment was against blank. Standard D,L alanine, and the amino acids were measured in μg alanine/min/g.b.wt.

#### 4.8.4. Phenol Oxidase Activity

The activity of phenol oxidase was measured according to a modification of [58]. Reaction mixture: phosphate buffer (0.1 M, pH 7) 0.5 mL, enzyme solution 200 µL, and catechol solution (2%) 200 µL. Enzyme reaction was initiated by adding catechol solution after 1 min, the optical density (O.D.) was determined, zero adjustment was against blank. The phenol oxidase activity was determined as O.D. units ×10^3^ at an absorbance of λ 405 nm

#### 4.8.5. Statistical Analysis

Mortality data were recorded in a probit regression line and we calculated LC_50,_ LC_90,_ slope function, and X^2^ [59], and correction for control mortality was conducted using Abbott’s formula according to [60]:

$$\%\;\mathrm{corrected}\;\mathrm{mortility}=\frac{\%\;\mathrm{mortility}\;\mathrm{in}\;\mathrm{treatment}-\%\;\mathrm{mortality}\;\mathrm{in}\;\mathrm{untreated}}{100-\%\;\mathrm{mortality}\;\mathrm{in}\;\mathrm{untreated}}\;\times \;100$$

## 5. Conclusions

The present study has shown that scopularide A exhibits larvicidal activity against third-instar larvae of *C. pipiens* with LC_50_ values of 69.963 and 58.968 after 72 and 96 h, respectively. The efficacy demonstrated could be encouraging evidence to enhance the existing methods in the vector mosquito control toolkit. The findings of this study reveal that scopularide A is a strong, biodegradable, and natural mosquito larvicidal agent that can be exploited to develop eco-friendly larvicides.

## Figures and Tables

**Table 1:** ^1^H-NMR, ATP, COSY, and HMBC spectral data of compound **1**.

Position	δ_C_ (ppm)	δ_H_ (ppm), Multiplicity, and *J* (Hz)	COSY	HMBC	Position	δ_C_ (ppm)	δ_H_ (ppm), Multiplicity, and *J* (Hz)	COSY	HMBC
Phenylalanine					Valine				
**1**	172.66	--			**1**	171.79	--		
**2**	53.25	4.83 (m)	3	1, 3, 4, Ala-1	**2**	57.93	4.21 (m)	3	1, 3, 4, 5
**3**	38.55	3.12 (d, *J* = 8.1 Hz) 3.09 (d, *J* = 8.1 Hz)	2	1, 2, 4, 5/9	**3**	31.11	2.18 (m)	2, 4, 5	1, 2, 4, 5
**4**	135.76	--			**4**	18.21	0.93 (d, *J* = 3.5 Hz)	3	2, 3, 5
**5/9**	129.10	7.16 (m)	6/8	3, 5/9, 7	**5**	19.60	0.93 (d, *J* = 3.5 Hz)	3	2, 3, 4
**6/8**	128.52	7.28 (m)	5/9	4, 6/8	**Glycine**				
**7**	127.07	7.21 (m)	6/8	5/9	**1**	171.17	--		
**Alanine**					**2**	43.14	3.44 (dd, *J* = 17.2, 4.1 Hz) 4.43 (d, *J* = 8.0 Hz)		1, HMDA-1
**1**	172.70	--			**Hydroxy methyl decanoic acid (HMDA)**				
**2**	49.53	4.24 (m)	3	1, 3, Leu-1	**1**	172.81	--		
**3**	17.50	1.29 (d, *J* = 7.3 Hz)	2	2, 3	**2**	40.98	2.40 (m)	3	
**Leucine**					**3**	78.14	4.64 (m)	2, 4	
**1**	173.28	--			**4**	37.92	1.58 (m)	3, 5, 11	3, 5, 6, 11
**2**	54.00	4.16 (m)	3		**5**	32.12	0.91 (m) 1.21 (m)	4, 6	3, 4, 7, 11
**3**	39.15	1.58 (m)	2,4		**6**	29.24	1.21 (m)	5, 7	4, 8
**4**	24.66	1.68 (m)	3, 5, 6		**7**	29.06	1.21 (m)	6, 8	5, 6, 8, 9
**5**	22.43	0.95 (d, *J* = 2.5 Hz)	4		**8**	33.20	1.28 (m)	7, 9	6, 7, 9, 10
**6**	22.01	0.91 (d, *J* = 2.2 Hz)	4		**9**	22.69	1.22 (m)	8, 10	7, 8, 10
					**10**	13.92	0.85 (t)	9	8, 9
					**11**	13.52	0.78 (d, *J* = 6.7 Hz)	4	3, 4, 5

**Table 2:** ^1^H-NMR, ATP, and COSY spectral data of compound **2**.

Position	δ_C_ (ppm)	δ_H_ (ppm), Multiplicity, and *J* (Hz)	HMBC	Position	δ_C_ (ppm)	δ_H_ (ppm), Multiplicity, and *J* (Hz)	HMBC
Phenylalanine				Valine			
**1**	172.29	--		**1**	171.81	--	
**2**	53.49	4.72–4.64 (m)	1, 3, 4, Ala-1	**2**	58.05	4.21 (t)	1, 3, 4, 5
**3**	40.93	2.98 (dd, *J* = 13.5, 7.5 Hz) 3.12 (dd, *J* = 13.5, 8.5 Hz)	1, 2, 4, 5/9	**3**	28.25	2.27 (m)	1, 2, 4, 5
**4**	135.74	--		**4**	18.39	0.97 (d, *J* = 2.3 Hz)	2, 3, 5
**5/9**	129.13	7.17 (m)	3, 5/9, 7	**5**	19.65	0.97 (d, *J* = 2.3 Hz)	2, 3, 4
**6/8**	128.55	7.26 (m)	4, 6/8	**Glycine**			
**7**	127.09	7.22 (m)	5/9	**1**	1711.11	--	
**Alanine**				**2**	43.21	4.46 (d, *J* = 8.0 Hz) 3.47 (dd, *J* = 17.2, 4.0 Hz)	1, HMOA-1
**1**	172.64	--		**Hydroxy methyl lactonic acid (HMOA)**			
**2**	49.43	4.14 (q)	1, 3, Leu-1	**1**	172.91	--	
**3**	17.89	1.29 (d, *J* = 7.3 Hz)	2,3	**2**	41.2	2.41(m)	
**Leucine**				**3**	77.98	4.80 (m)	2, 5, 9
**1**	172.94	--		**4**	37.74	1.50 (m) 1.2 (m)	3, 5, 6, 9
**2**	53.23	4.27 (t)	1, 3, 4, Val-1	**5**	32.48	1.19 (m) 0.91 (m)	3, 4, 7, 9
**3**	39.06	1.60–1.57 (m)	1, 2, 4, 5, 6	**6**	29.34	1.10 (m)	4, 7, 8
**4**	24.52	1.64 (m)	2, 3, 5, 6	**7**	22.59	1.24 (m)	5, 6, 8
**5**	22.02	0.97 (d, *J* = 2.2 Hz)	3, 4, 6	**8**	13.67	0.87 (t)	6, 7
**6**	22.55	0.95 (d, *J* = 2.2 Hz)	3, 4, 5	**9**	14.05	0.75 (d, *J* = 6.7 Hz)	3, 4, 5

**Table 3:** Susceptibility of third-instar larvae *C. pipiens* to isolated compounds at different time intervals.

	Percentage Mortality (%)							
Concentrations (ppm)	Scopularide A				Scopularide B			
	24 h	48 h	72 h	96 h	24 h	48 h	72 h	96 h
**300**	21.33	25.33	80	82.66	20	24	78.66	80
**200**	17.33	20	69.33	72	16	18.66	68	70.66
**100**	13.33	16	53.33	56	12	14.66	52	53.33
**50**	10.66	13.33	34.66	37.33	9.33	12	33.33	36
**10**	9.33	12	24	26.66	8	10.66	22.66	25.33
**LC_50_ (ppm)**	74,714.96	51,225.03	69.96	58.96	65,489.96	50,583.51	76.17	66.14
**LC_90_ (ppm)**	254,765,445.4	306,771,168.1	1167.2097	994.31	121,222,443.6	183,614,892.8	1256.21	1180.08
**Slope ± SE**	0.3628 ± 0.14	0.3393 ± 0.13	1.0485 ± 0.1203	1.0446 ± 0.1193	0.3922 ± 0.1493	0.36 ± 0.13	1.0529 ± 0.12	1.0241 ± 0.11

**Table 4:** Susceptibility of third-instar larvae *C. pipiens* crude extracts of *A. flavus and A. nomius* at different time intervals.

	Percentage Mortality (%)							
Conc (ppm)	*A. nomius* Extract				*A. flavus* Extract			
	24 h	48 h	72 h	96 h	24 h	48 h	72 h	96 h
**300**	18.66	22.66	76	78.66	17.33	20	73.33	74.66
**200**	14.66	17.33	66.66	69.33	13.33	14.66	64	65.33
**100**	10.66	13.33	49.33	49.33	9.33	12	46.66	48
**50**	8	10.66	32	34.66	6.66	8	29.33	30.66
**10**	6.66	9.33	20	24	5.33	6.66	17.33	18.66
**LC_50_ (ppm)**	52,659.3	46,847.74	87.2	74.3	40,102.7	32,051.5	102.8	94.8
**LC_90_ (ppm)**	49,875,033.8	98,192,084.9	1366.5	1328.8	19,084,973.0	20,417,679.0	1522.9	1449.9
**Slope ± SE**	0.4306 ± 0.15	0.3859 ± 0.14	0.1437 ± 0.12	1.0232 ± 0.12	0.4787 ± 0.16	0.457 ± 0.15	1.0947 ± 0.12	1.0818 ± 0.12

**Table 5:** Effect of LC_50_ values of the tested samples on chitinase activity of *C. pipiens* larvae.

Sample	(mg *N-*Acetylglucoseamine/min/mg Protein) Mean ± SD	% Change
***A. flavus*** **crude extract**	118 ± 8.6 ^a^	9.3
***A. nomius*** **crude extract**	95 ± 3.2 ^b^	−12.0
**Scopularide B**	86 ± 2.5 ^b^	−20.4
**Scopularide A**	82 ± 2.4 ^b^	−24.1
**Control**	108 ± 5.1 ^a^	

Means bearing different scripts are significantly different from control at *p* < 0.01 (Duncan’s multiple range test).

**Table 6:** Effect of LC_50_ values of the tested samples on phenoloxidase activity of *C. pipiens*.

Sample	(O.D. Units ×10^3^/min/mg Protein) Mean ± SD	% Change
***A. flavus*** **crude extract**	1579 ± 14.5 ^c^	−11.88
***A. nomius*** **crude extract**	1772 ± 15.9 ^a^	−1.11
**Scopularide B**	1598 ± 13 ^bc^	−10.82
**Scopularide A**	1631 ± 9 ^b^	−8.98
**Control**	1792 ± 15.6 ^a^	

Means bearing different scripts are significantly different from control at *p* < 0.01 (Duncan’s multiple range test).

**Table 7:** Effect of LC_50_ values of the tested samples on lipase activity of *C. pipiens* larvae.

Sample	(µM Oleic Acid Liberated/min/gram Body Weight) Mean ± SD	% Change
***A. flavus*** **crude extract**	4.6 ± 0.22 ^a^	−52.7
***A. nomius*** **crude extract**	2.9 ± 0.1 ^c^	0.3
**Scopularide B**	3.8 ± 0.16 ^b^	−26
**Scopularide A**	3.9 ± 0.15 ^b^	−28.3
**Control**	3 ± 0.12 ^c^	

Means bearing different scripts are significantly different from control at *p* < 0.01 (Duncan’s multiple range test).

**Table 8:** Effect of LC_50_ values of the tested samples on protease activity of *C. pipiens* larvae.

Sample	(ng D,L Alanine/min/mg Protein) Mean ± SD	% Change
***A. flavus*** **crude extract**	3235 ± 106 ^ab^	5.6
***A. nomius*** **crude extract**	2873 ± 61 ^c^	16.1
**Scopularide A**	3116 ± 78 ^b^	9.0
**Scopularide B**	2277 ± 42 ^d^	33.5
**Control**	3426 ± 88 ^a^	

Means bearing different scripts are significantly different from control at *p* < 0.01 (Duncan’s multiple range test).

## Supplementary Materials

The following supporting information can be downloaded at: https://www.mdpi.com/article/10.3390/md20050331/s1, Table S1. ^1^H-NMR and ATP spectral data of compound **1** [29], Table S2. ^1^H-NMR and ATP spectral data of compound **2 [29]**.
