# Supplementary material for: Cyclodepsipeptides: Isolation from Endophytic Fungi of Sarcophyton ehrenbergi and Verification of Their Larvicidal Activity via In-Vitro and In-Silico Studies
Source: Mar Drugs. 2022 May 18;20(5):331. doi: 10.3390/md20050331 (PMC9146806; doi:10.3390/md20050331)
Supplement: Supplementary file 1 [file marinedrugs-20-00331-s001.zip › marinedrugs-1709203-supplementary.pdf]

Supplementary file

Article

Cyclodepsipeptides: Isolation from Endophytic Fungi of *Sarcophyton ehrenbergi* and Verification of Their Larvicidal Activity via In-Vitro and In-Silico Studies

Table S1. ^{1}H-NMR, and ATP spectral data of compound **1**.

Position	δ_C (ppm)	δ_H (ppm), multiplicity and J (Hz)	*Ref. δ_C (ppm)	*Ref. δ_H (ppm)	Position	δ_C (ppm)	δ_H (ppm), multiplicity and J (Hz)	*Ref. δ_C (ppm)	*Ref. δ_H (ppm)
**Phenylalanine**					**Valine**				
**1**	172.66	--	173.6		**1**	171.79	--	173.4	
**2**	53.25	4.83 (m)	55.3	4.79, m	**2**	57.93	4.21, m	59.9	4.20, m
**3**	38.55	3.12 (d, J = 8.1 Hz)<br>3.09 (d, J = 8.1 Hz)	39.4	3.18, d<br>3.03, d	**3**	31.11	2.18(m)	31.0	2.19, m
**4**	135.76	--	137.8		**4**	18.21	0.93 (d, J = 3.5 Hz)	19.0	0.98, d
**5/9**	129.10	7.16 (m)	130.3	7.24, m	**5**	19.60	0.93 (d, J = 3.5 Hz)	19.7	0.97, d
**6/8**	128.52	7.28 (m)	129.6	7.28, m	**Glycine**				
**7**	127.07	7.21 (m)	128.0	7.21, m	**1**	171.9		171.9	
**Alanine**					**2**	43.14	3.44 (dd, J = 17.2, 4.1 Hz)<br>4.43 (d, J = 8.0 Hz)	44.0	4.24,d<br>3.53, d
**1**	172.70	--	174.1		**Hydroxy methyl decanoic acid (HMDA)**				
**2**	49.53	4.24 (m)	50.3	4.29, q	**1**	172.81	--	174.5	
**3**	17.50	1.29 (d, J = 7.3 Hz)	17.6	1.27, d	**2**	40.98	2.40 (m)	40.9	2.42, m
**Leucine**					**3**	78.14	4.64 (m)	78.6	4.77, m
**1**	173.28	--	175.1		**4**	37.92	1.58 (m)	39.0	1.54, m
**2**	54.00	4.16 (m)	54.7	4.19, m	**5**	32.12	0.91(m)<br>1.21 (m)	33.6	1.22.m<br>0.95, m
**3**	39.15	1.58 (m)	40.3	1.58, m	**6**	29.24	1.21 (m)	30.6	1.30, m
**4**	24.66	1.68 (m)	26.0	1.68, m	**7**	29.06	1.21 (m)	28.2	1.29, m

**5**	22.43	0.95 (d, *J* = 2.5 Hz)	23.1	1.00, d	**8**	33.20	1.28 (m)	33.0	1.26, m
**6**	22.01	0.91 (d, *J* = 2.2 Hz)	22.3	0.95, d	**9**	22.69	1.22 (m)	23.7	1.31, m
					**10**	13.92	0.85 (t)	14.42	0.91, t
					**11**	13.52	0.78 (d, *J* = 6.7 Hz).	14.41	0.83, d

* These reference data are according to reported data by Yu et al. [29].

Table S2. ^{1}H-NMR and ATP spectral data of compound **2**.

**Position**	**δC (ppm)**	**δH (ppm), multiplicity and J (Hz)**	***Ref. δC (ppm)**	***Ref. δH (ppm)**	**Position**	**δC (ppm)**	**δH (ppm), multiplicity and J (Hz)**	***Ref. δC (ppm)**	***Ref. δH (ppm)**
**Phenylalanine**					**Valine**				
1	172.29	--	173.6	--	1	171.81	--	173.4	
2	53.49	4.72 – 4.64 (m)	55.3	4.79, m	2	58.05	4.21 (t)	59.9	4.20, m
3	40.93	2.98 (dd, J = 13.5, 7.5 Hz)<br>3.12 (dd, J = 13.5, 8.5 Hz)	39.4	3.18, dd<br>3.03, dd	3	28.25	2.27 (m)	31.0	2.18, m
4	135.74	--	137.8	--	4	18.39	0.97 (d, J = 2.3 Hz)	19.0	0.98, d
5/9	129.13	7.17 (m)	130.3	7.24, m	5	19.65	0.97 (d, J = 2.3 Hz)	19.7	0.97, d
6/8	128.55	7.26 (m)	129.6	7.28, m	**Glycine**				
7	127.09	7.22 (m)	128.0	7.21, m		171.1	--	171.9	
**Alanine**					2	43.21	4.46 (d, J = 8.0 Hz)<br>3.47 (dd, J = 17.2, 4.0 Hz)	44.0	4.24,d<br>3.53, d
1	172.64	--	174.1		**Hydroxy methyl lactonic acid (HMOA)**				
2	49.43	4.14 (q)	50.3	4.29, q	1	172.91	--	174.5	
3	17.89	1.29 (d, J = 7.3 Hz)	17.6	1.28, d	2	41.2	2.41(m)	40.9	2.41, m
**Leucine**					3	77.98	4.80 (m)	78.6	4.77, m
1	172.94	--	175.1		4	37.74	1.50 (m)<br>1.2 (m)	39.0	1.54m<br>1.22 m
2	53.23	4.27 (t)	54.7	4.19, m	5	32.48	1.19 (m)<br>0.91 (m)	33.3	0.96 m<br>1.32 m
3	39.06	1.60 – 1.57 (m)	40.4	1.58, m	6	29.34	1.10 (m)	30.5	1.32 m
4	24.52	1.64 (m)	26.0	1.69, m	7	22.59	1.24 (m)	23.8	1.16 m
5	22.02	0.97 (d, J = 2.2 Hz)	23.1	0.99, d	8	13.67	0.87 (t)	14.38	0.90 t
6	22.55	0.95 (d, J = 2.2 Hz)	22.3	0.95, d	9	14.05	0.75 (d, J = 6.7 Hz)	14.41	0.83 d

* This reference data are according to reported data by Yu et al. [29].

Reference

Yu, Z.; Lang, G.; Kajahn, I.; Schmaljohann, R.; Imhoff, J.F. Scopularides A and B, Cyclodepsipeptides from a Marine Sponge-Derived Fungus, *Scopulariopsis brevicaulis*. *J. Nat. Prod.* **2008**, *71*, 1052–1054.
